# Computed Tomography-Based Radiomics Model to Predict Central Cervical Lymph Node Metastases in Papillary Thyroid Carcinoma: A Multicenter Study

**DOI:** 10.3389/fendo.2021.741698

**Published:** 2021-10-21

**Authors:** Jingjing Li, Xinxin Wu, Ning Mao, Guibin Zheng, Haicheng Zhang, Yakui Mou, Chuanliang Jia, Jia Mi, Xicheng Song

**Affiliations:** ^1^ Second Clinical Medicine College, Binzhou Medical University, Yantai, China; ^2^ Department of Otorhinolaryngology-Head and Neck Surgery, Yantai Yuhuangding Hospital, Qingdao University, Yantai, China; ^3^ Department of Radiology, Yantai Yuhuangding Hospital, Yantai, China; ^4^ Big Data and Artificial Intelligence Laboratory, Yantai Yuhuangding Hospital, Yantai, China; ^5^ Shandong Provincial Clinical Research Center for Otorhinolaryngologic Diseases, Yantai, China; ^6^ Department of Thyroid Surgery, Yantai Yuhuangding Hospital, Yantai, China; ^7^ Precision Medicine Research Center, Binzhou Medical University, Yantai, China

**Keywords:** computer aided diagnosis, machine learning, radiomics, central lymph node metastases, papillary thyroid carcinoma

## Abstract

**Objectives:**

This study aimed to develop a computed tomography (CT)-based radiomics model to predict central lymph node metastases (CLNM) preoperatively in patients with papillary thyroid carcinoma (PTC).

**Methods:**

In this retrospective study, 678 patients with PTC were enrolled from Yantai Yuhuangding Hot3spital (n=605) and the Affiliated Hospital of Binzhou Medical University (n=73) within August 2010 to December 2020. The patients were randomly divided into a training set (n=423), an internal test set (n=182), and an external test set (n=73). Radiomics features of each patient were extracted from preoperative plain scan and contrast-enhanced CT images (arterial and venous phases). One-way analysis of variance (ANOVA) and least absolute shrinkage and selection operator algorithm were used for feature selection. The K-nearest neighbor, logistics regression, decision tree, linear-support vector machine (linear-SVM), Gaussian-SVM, and polynomial-SVM algorithms were used to establish radiomics models for CLNM prediction. The clinical risk factors were selected by ANOVA and multivariate logistic regression. Incorporated with clinical risk factors, a combined radiomics model was established for the preoperative prediction of CLNM in patients with PTCs. The performance of the combined radiomics model was evaluated using the receiver operating characteristic (ROC) and calibration curves in the training and test sets. The clinical usefulness was evaluated through decision curve analysis (DCA).

**Results:**

A total of 4227 radiomic features were extracted from the CT images of each patient, and 14 non-zero coefficient features associated with CLNM were selected. Four clinical variables (sex, age, tumor diameter, and CT-reported lymph node status) were significantly associated with CLNM. Linear-SVM led to the best prediction model, which incorporated radiomic features and clinical risk factors. Areas under the ROC curves of 0.747 (95% confidence interval [CI] 0.706–0.782), 0.710 (95% CI 0.634–0.786), and 0.764 (95% CI 0.654–0.875) were obtained in the training, internal, and external test sets, respectively. The linear-SVM algorithm also showed better sensitivity (0.702 [95% CI 0.600–0.790] *vs.* 0.477 [95% CI 0.409–0.545]) and accuracy (0.670 [95% CI 0.600–0.738] *vs.* 0.642 [95% CI 0.569–0.712]) than an experienced radiologist in the internal test set in the combined radiomics model. The calibration plot reflected a favorable agreement between the actual and estimated probabilities of CLNM. The DCA indicated the clinical usefulness of the combined radiomics model.

**Conclusion:**

The combined radiomics model is a non-invasive preoperative tool that incorporates radiomic features and clinical risk factors to predict CLNM in patients with PTC.

## Introduction

Papillary thyroid carcinoma (PTC) is the predominant pathological type of thyroid malignancy (1), and its incidence has rapidly increased in the past decade (2, 3). Although PTC is regarded as an indolent tumor, a portion of cancer cells metastasize to lymph nodes (LNs) around the thyroid gland, including central cervical lymph node metastases (CLNM) and lateral CLNM (4). CLNM has been reported in up to 60%–70% of patients and is an important risk factor for locoregional recurrence (5–9). CLNM is an independent risk factor for the prognosis of patients with PTC (10). Tumor cells usually occur first in the central neck (level VI) and then in the lateral neck (levels II, III, and IV) (11). LN metastases rarely occur first in the lateral neck compartments (12, 13). Therefore, central cervical compartment has the highest risk of metastases (14). Central cervical LNs are the “sentinel” LNs of PTC neck LNs. Improving the diagnostic level of central cervical LNs will help reduce unnecessary LN dissection. However, how to identify patients with CLNM accurately remains a problem because of the lack of discriminate features. Preoperative diagnosis of CLNM plays an important role in the formulation of surgical plans (15–17). Thus, the accurate preoperative prediction of CLNM provides a reference for individualized treatment decision.

Currently, ultrasound (US) and contrast-enhanced computed tomography (CT) plays an important role in preoperative nodal staging and is a standard procedure in clinical practice (18, 19). However, preoperative US can only detect 20%–31% of CLNM (20, 21) and may only change the surgical procedure of 20% patients (22). Its efficacy in identifying malignant nodes is unsatisfactory (23). By contrast, CT has a variable sensitivity of 35%–77% and specificity of 70%–96% for the diagnosis of CLNM in patients with thyroid cancer (24, 25). Meanwhile, the diagnostic efficacy of CT is still limited by the subjective judgment of different radiologists, which may lead to a considerable proportion of patients being under-staged or over-staged.

Radiomics have recently been introduced into medical imaging for preoperative diagnosis as computer-aided systems. It refers to the high-throughput extraction of large numbers of imaging features, thus converting medical images into mineable high-dimensional data; the subsequent quantitative analysis of these data can be used to support surgical decision making (26, 27). Radiomics-based methods were also proposed for the prediction of LN metastases in patients with PTC by converting radiological images into minable high-dimensional data (28–30). To our knowledge, some radiomics-based studies have focused on the preoperative prediction of CLNM in patients with PTC to date, but most of these studies are based on US images to develop radiomics models (31, 32) and are single centered. Studies have yet to develop a radiomics-based model with CT images among multicenter to predict CLNM in patients with PTC.

Hence, the study aimed to develop a CT-based radiomics model that would incorporate radiomics features and clinical risk factors for the preoperative prediction of CLNM in patients with PTC and being tested in different centers. This comprehensive model may assist clinicians in selecting the most appropriate treatment strategies to achieve a better outcome.

## Materials and Methods

### Patients

This retrospective study was approved by the Ethics Committees of Yantai Yuhuangding Hospital and the Affiliated Hospital of Binzhou Medical University, exempted from informed consent in accordance with the Helsinki declaration. This analysis was performed on patients who underwent surgical treatment and were pathologically diagnosed with PTC in Yantai Yuhuangding Hospital (denote as Hospital 1) and the Affiliated Hospital of Binzhou Medical University (denote as Hospital 2) from August 2010 to December 2020. The inclusion criteria were as follows: (a) single lesion; (b) histologically confirmed with PTC; (c) underwent no preoperative anticancer treatment; (d) contrast-enhanced CT was performed within 2 weeks before surgery; and (e) patients underwent neck dissection with ipsilateral lobectomy or total thyroidectomy and received pathological diagnosis of LNs. The exclusion criteria were as follows: (a) postoperative pathological examination showed concomitant non-PTC components in the lesion (such as atypical hyperplasia, follicular tumors, medullary carcinomas, undifferentiated carcinomas, and metastatic carcinomas); (b) biopsy before CT scan; (c) without preoperative thyroid CT scan; (d) the patient was suffering from other concomitant malignancies, such as lymphoma, breast cancer, or liver cancer; (e) the primary tumor was unclear on CT images; (f) the primary tumor had maximum diameter < 0.5 cm; (g) the primary tumor was difficult to segment because of nodular goiter or chronic lymphocytic thyroiditis; and (h) postoperative pathological examination showed multifocal PTC.

A total of 678 patients from two hospitals were included in this study. Patients in Hospital 1 (N=605) were divided into a training set (N=423) and an internal test set (N=182) at a ratio of 7:3 by using a completely random classification method. Patients in Hospital 2 were included as an external test set (N=73). Clinical data of patients were collected, including age, sex, CT primary tumor maximum diameter, location, and CT-reported LN status.

Preoperative CT findings on LN status were used as the CT-reported LN status. In accordance with the National Comprehensive Cancer Network (version3.2020) Guidelines and some previous studies (19, 23, 33, 34), metastatic lymph nodes (LN+) were considered when at least one of the following criteria presented: (a) LN ≥ 10 mm in the maximal short axis diameter; (b) round or irregular shape; (c) rough margin, fuzzy boundary, and/or invasion into adjacent tissues; (d) calcification or cystic and/or necrotic change; (e) strong enhancement (similar to or stronger than that of the pharyngeal mucosa); and (f) heterogeneous enhancement. On the basis of previous studies (35, 36), suspicious LNs (LN_suspicious_) were considered when the LNs did not meet the above criteria but were ≥5 mm in the maximal short axial diameter at cervical level VI. CT-reported LN status was assessed by a radiologist (Dr. A) with 12 years of experience in thyroid radiology, and the results were verified by another radiologist (Dr. B) who had 22 years of subspecialty experience in thyroid radiology. The weighted kappa coefficient was used to evaluate the agreement between these two radiologists. For assessment of overall agreement, the mean κ value was calculated from these pairs. Strength of κ agreement was defined as follows: < 0.00, poor; 0.00–0.20, slight; 0.21–0.40, fair; 0.41–0.60, moderate; 0.61–0.80, substantial; and 0.81–1.00, almost perfect (37). All disagreements were resolved through consultation.

### Surgery Strategy

Patients received lobectomy and isthmectomy or total thyroidectomy depending on the clinical TNM stage. For patients with lateral cervical LN metastases, lateral cervical LN dissection was performed. The resected thyroid tissues were processed for pathological examination (including the determination of unifocal or multifocal PTC). The resected LNs were also subjected to pathological examination, and LN metastases were determined.

### CT Images Acquisition

Before receiving surgery, all patients underwent contrast-enhanced CT on thyroid with a 16-slice spiral CT scanner (Sensation 16; SIEMENS) or a 64-slice spiral CT scanner (Sensation 64; SIEMENS). After plain CT scanning, a dynamic contrast-enhanced CT scan was performed after intravenous administration of 80–100 mL nonionic contrast material (Iopamidol, 370 mg I/mL, Bracco, Milan, Italy) by using power injection at a rate of 3.5 mL/s followed by saline flush (20 mL). Arterial and venous phase images were obtained at 25 and 60 s, respectively. The slice thickness of the reconstructed image was 1.0 mm. Arterial phase, venous phase, and plain scan CT images were retrieved for image feature extraction. All lesion image parameters (primary tumor maximum diameter, location) were examined and assessed by radiologist Dr. A, and the results were verified by another radiologist Dr. B. The CT images of all target lesions were exported from the Picture Archiving and Communicating System with Digital Imaging and Communications in Medicine format and imported into Radcloud (Huiying Medical Technology Co., Ltd.).

### CT Images Preprocessing and Segmentation

**Figure 1:**
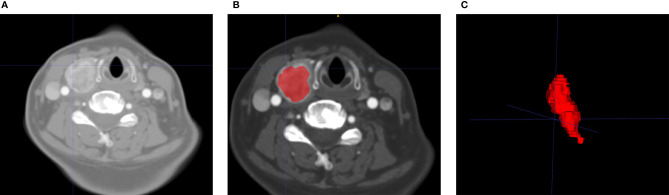
Flowchart of the process of CT image segmentation and VOI construction. An example of primary tumor segmentation and VOI construction for patients with central lymph node metastasis. VOI segmentation is performed on unenhanced and contrast-enhanced computed tomography images **(A)**. Features are extracted from the VOI of primary tumor **(B)**, including tumor shape, intensity, and texture. The VOI of primary tumor can be shown in a 3D status **(C)**. VOI, volume of interest.

Al CT images were imported into Radcloud for image preprocessing, including image normalization, to reduce the difference in grayscale texture between images. The data were resampled to 1 mm*1 mm*1 mm to eliminate the difference in different image scales. Before feature selection, tumor segmentation was performed by manually delineating the volume of interest (VOI) along the tumor contour on each CT image slice using Radcloud. Radiologist Dr. A (12 years of experience) used Radcloud to manually delineate the VOI of the primary thyroid tumor as consistent as possible with the margins of the primary tumor lesion. This study did not delineate the LN region but the primary thyroid tumor because many metastatic LNs are extremely small and difficult to identify on the CT image. In addition, many LNs were unable to correspond to each other between CT image and pathology. For these reasons, this prediction model was based on the primary thyroid tumor. The primary tumor VOI region was evaluated by radiologist Dr. B (22 years of subspecialty experience) to ensure the accuracy of segmentation. Following the same guideline describing how to define the boundary of tumors, both doctors were aware of the diagnosis of thyroid cancer but were blinded to the clinical and histopathologic data. The schematic of image segmentation is shown in **Figure 1**.

### Feature Extraction and Selection

Radiomic features (shape, first-order, texture features) were extracted from each VOI by Radcloud. All feature extraction methods were implemented in Radcloud. These features were divided into three groups, namely, first-order statistics, shape features, and texture features. Intra class correlation coefficients (ICCs) were utilized for evaluating the intra- and inter-observer agreement in terms of feature extraction. First, Dr. A and Dr. B randomly selected the images of 30 patients to evaluate the inter-class reproducibility. After a month, Dr. A repeated the same procedure. The remaining VOI segmentation was performed by Dr. A. Features with ICCs greater than 0.75 indicated satisfactory reproducibility and were selected for radiomic features, and those with ICCs less than 0.75 were eliminated. After one-way analysis of variance (ANOVA), features with *p <*0.05 were selected. Then, the Least Absolute Shrinkage and Selection Operator (LASSO) algorithm was used to select non-zero coefficients. The optimal penalization coefficient alpha was set by five-fold cross-validation, and radiomic features with non-zero coefficients within the training set were finally selected. To avoid overfitting, we ensured the feature selection, and the subsequent classifications were performed only on the training set rather than on the entire dataset.

### Radiomics Model Construction

Basing the radiomic features that were selected from the training set, this study used six algorithms to construct the radiomics model for CLNM prediction: K-nearest neighbor (KNN), logistic regression (LR), decision tree (DT), linear-support vector machine (linear-SVM), Gaussian-SVM, and polynomial-SVM. Linear-SVM, Gaussian-SVM, and polynomial-SVM are SVM algorithms with different kernels, which may cause different results.

### Clinical Independent Risk Factor Selection

The significant clinical risk factors were determined using ANOVA. Then, the significant clinical risk factors were integrated into the multivariate LR analysis of backward-stepwise selection. During this procedure, collinearity was considered and the factors with variance inflation factor (VIF) > 10 and *p* value > 0.05 were eliminated. Akaike information was used as the criterion when the smallest Akaike information criterion was reached, the stepwise procedure was stopped, and the final multivariate LR selected the independent clinical factors.

### Construction of the Combined Radiomics Model

The six algorithms (KNN, LR, DT, linear-SVM, Gaussian-SVM, and polynomial-SVM) were used to construct combined radiomics models, which cooperate selected radiomics features and independent clinical risk factors, and the best combined model was selected.

### Assessment and Test of Combined Radiomics Model Performance

The prediction performance of the combined radiomics models in the training and test sets was evaluated using the area under the receiver operator characteristic (ROC) curve (AUC). The Youden index method was used to identify the optimal cut-off value in ROC curves. Sensitivity, specificity, and accuracy values were determined by the cut-off value. Calibration of the model was assessed using Platt method (38). The calibration curve of the prediction model is an important index to evaluate the probability accuracy of a disease risk model to predict an individual outcome event in the future. A good degree of calibration indicates a high accuracy of the prediction model, whereas a poor degree of calibration suggests that the model may overestimate or underestimate the risk of disease. Good agreement between the true state of CLNM and the predicted probability based on the radiomics model was achieved when the calibration curves were close to the diagonal line. Decision curve analysis (DCA) was used to evaluate the impact of the radiomics model on the patients’ benefit in predicting the CLNM of PTC to assist clinicians in making treatment decisions. Probability thresholds are important for the trade-off between accepting necessary clinical procedures and avoiding unnecessary ones. The curve with the highest gain is the best prediction method.

### Statistical Analysis

All statistical tests were conducted in Python 3.6, R software 4.0.3, and SPSS 26. Scikit-Learn, a python library, was performed for the radiomic feature selection and model construction. The modules of “feature-selection”, “linear-model”, “svm”, “neighbors”, “tree”, and “metrics” were used in feature selection and model construction. In R software, the “rms” package was used to select clinical variables and plot the calibration curves. The “rmda” package was used to perform DCA. In SPSS, categorical variables (i.e., sex and CT-reported LN status) were compared using the χ^2^ test or Fisher exact test. Continuous variables (i.e., age, tumor diameter) were compared using Student’s t test or Mann-Whitney U test, when appropriate. All levels of statistical significance were two-sided, and *p <*0.05 was considered to indicate statistical significance. DeLong’s test was used to compare the differences in ROC curves.

## Results

### Demographics Features

**Figure 2:**
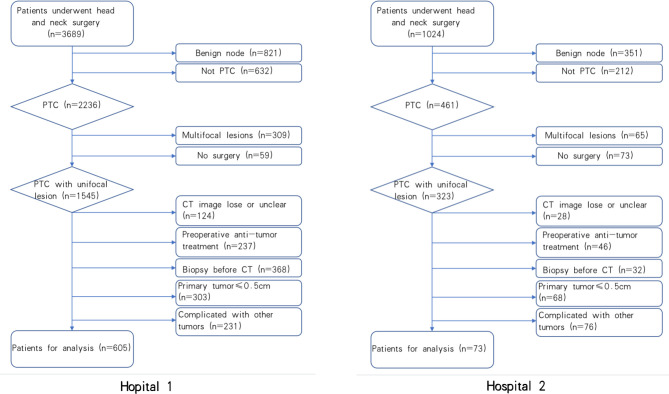
The flow chart of patients Recruitment process. Hospital 1 stands for patients from Yantai Yuhuangding Hospital, Hospital 2 stands for patients from the Affiliated Hospital of Binzhou Medical University.

The flow chart of patient screening is shown in **Figure 2**. From August 2010 to December 2020, 3689 candidates at Hospital 1 were identified, and 605 who met the inclusion criteria were enrolled and divided into a training set (mean age ± standard deviation [SD], 44±12) and an internal test set (mean age ± standard deviation [SD], 44±12). Similarly, 73 patients were enrolled from August 2010 to December 2020 at Hospital 2 (external test set, mean age ± SD, 44 ±11). The clinical characteristics of 678 patients with PTC were summarized. Of the 678 eligible patients, their average age was 44 ± 12 years. The male-to-female patient ratio was 1:2.8. The mean tumor size was 1.18 cm (median=1.0 cm). The weighted kappa coefficient in CT-reported LN status was 0.8. The clinical information of patients in the training set, internal test set, and external test set is shown in **Table 1**.

**Table 1:** Clinical characteristics of patients in the training and test sets.

Characteristics	Training Set (N = 423)			Internal test set (N = 182)			External test set (N = 73)		
	CLNM (+) (N = 218)	CLNM (-) (N = 205)	p-value	CLNM (+) (N = 94)	CLNM (-) (N = 88)	p-value	CLNM (+) (N = 46)	CLNM (-) (N = 27)	p-value
Age, mean ± SD, years	44± 12	44 ± 12		44± 12	44 ± 12		44 ± 12	44 ± 12	
< 45, No. (%)	131 (60.09)	84 (40.98)	<0.001	52 (55.32)	41 (46.59)	0.239	21 (45.65)	12 (44.44)	0.92
≥ 45, No. (%)	87(39.91)	121 (59.02)		42 (44.68)	47 (53.41)		25 (54.35)	15 (55.56)	
Sex, No. (%)									
Male	70 (32.11)	45 (21.95)	<0.001	24 (25.53)	19 (21.59)	0.532	18 (39.13)	2 (28.57)	0.793
Female	148 (67.89)	160 (78.05)		70 (74.47)	69 (78.41)		28 (60.87)	25 (71.43)	
CT-maximum diameter*, No. (%)									
<1cm	88 (40.37)	120 (58.54)	<0.001	35 (37.23)	55 (62.50)	<0.05	19 (41.30)	12 (44.44)	0.793
≧1cm	130 (59.63)	85(41.46)		59 (62.77)	33 (37.50)		27 (58.70)	5 (55.56)	
CT-reported LN status, No. (%)									
LN-negative	114 (52.29)	147 (71.71)	<0.001	45 (47.87)	68 (77.27)	<0.05	10 (21.74)	4 (14.81)	0.468
LN-suspicious	39 (17.89)	45 (21.95)		23 (24.47)	12 (13.64)		0 (0.00)	0 (0.00)	
LN-positive	65 (29.82)	13 (6.34)		26 (27.66)	8 (9.09)		36 (78.26)	23 (85.19)	

Data are n (%) unless otherwise indicated. No significant divergences were found between CLNM (+) and CLNM (-) in terms of age, sex, CT-maximum diameter, CT-reported LN status in the training set (p < 0.05). CT-longest diameter and CT-reported LN status were significantly different between CLNM (+) and CLNM (-) in the internal test set (p < 0.05), but not in the external test set.

CT, computed tomography; LN, lymph node; CLNM, central lymph node metastasis.

*Largest diameter of the target primary lesion.

### Feature Selection and Radiomics Model Construction

**Figure 3:**
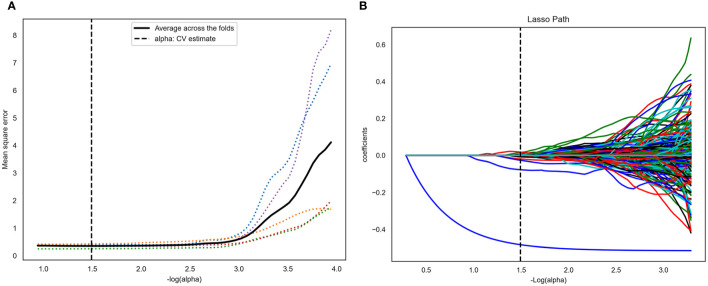
Computed tomography (CT) image features selection using one-way analysis of variance (ANOVA) and the least absolute shrinkage and selection operator (LASSO) logistic regression model in the training set. **(A)** The five-fold cross-validation and the minimal criteria process was used to generate the optimal penalization coefficient lambda (λ) in the LASSO model. The vertical line defines the optimal values of λ, where the model provides its best fit to the data. The optimal λ value of 0.165 with -log (λ) =1.5 was selected. **(B)** LASSO coefficient profiles of the radiomics features. The vertical line was drawn at the value selected using five-fold cross-validation, where optimal λ resulted in 14 nonzero coefficients.

**Figure 4:**
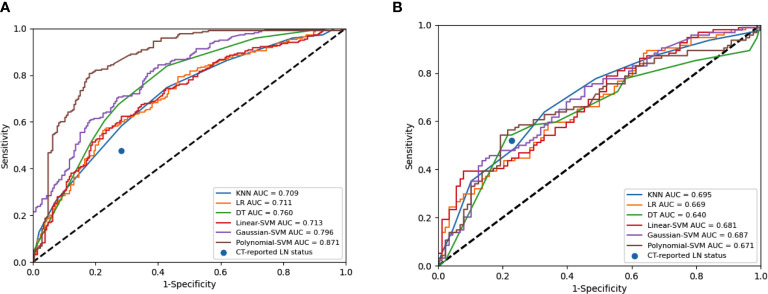
Receiver operating characteristic (ROC) curves of the radiomics model constructed by six algorithms in the training set **(A)** and internal test set **(B)**.

A total of 4227 radiomic features were extracted from the arterial phase, venous phase, and plain scan CT images of each patient. The ICCs ranged from 0.864 to 0.989 in the intra-observer and from 0.832 to 0.957 in the inter-observer. This result indicated that feature extraction within and between observers had good repeatability. Finally, 14 non-zero coefficient features (**Table 2**) associated with CLNM prediction were selected after ANOVA and LASSO (**Figures 3A, B**). In the training set, the polynomial-SVM algorithm demonstrated the highest values for AUC 0.871(95% [confidence interval (CI) 0.841–0.898), whereas the AUC values of KNN, LR, DT, linear-SVM, and Gaussian-SVM were 0.709 (95% CI 0.760–0.744), 0.711 (95% CI 0.669–0.748), 0.760 (95% CI 0.717–0.793), 0.713 (95% CI 0.671–0.751), and 0.796 (95% CI 0.760–0.827), respectively. In the internal test set, the KNN algorithm showed the highest values for AUC (0.695 [CI 0.584–0.699]), whereas the AUC values of LR, DT, linear-SVM, Gaussian-SVM, and polynomial-SVM were 0.669 (95% CI 0.522–0.646), 0.640 (95% CI 0.522–0.646), 0.681 (95% CI 0.533–0.656), 0.687 (95% CI 0.549–0.644), 0.671 (95% CI 0.526–0.647), respectively (**Figures 4** **A, B**).

**Table 2:** Radiomics features selected in ANOVA and LASSO regression analysis.

Radiomics features	Coefficient
lbp-2D_firstorder_Range_ scan	0.007418999
lbp-2D_firstorder_10Percentile_scan	0.004015608
Wavelet-HHL_glrlm_RunLengthNonUniformityNormalized_scan wavelet-HHH_ngtdm_Contrast_scan	-0.07654446 -0.00770341
wavelet-HLH_glszm_GrayLevelNonUniformityNormalized_scan	-0.01873799
wavelet-HHL_firstorder_10Percentile_scan	0.015804638
wavelet-HLH_glszm_SmallAreaHighGrayLevelEmphasis_scan	-0.02602846
wavelet-HHH_glszm_HighGrayLevelZoneEmphasis_scan	0.001441019
wavelet-HHL_firstorder_Mean_scan	0.003108156
original_shape_LeastAxisLength_AP	0.006855814
original_shape_Elongation_AP	0.00434467
original_gldm_LargeDependenceEmphasis_AP	0.017969827
original_glrlm_RunLengthNonUniformityNormalized_AP	-0.0047654
logarithm_glrlm_RunLengthNonUniformityNormalized_AP	-0.00065297

Fourteen radiomics features with non-zero coefficients in one-way analysis of variance (ANOVA) and the least absolute shrinkage and selection operator (LASSO) logistic regression model were selected.

### Development and Test of the Combined Radiomics Model

**Figure 5:**
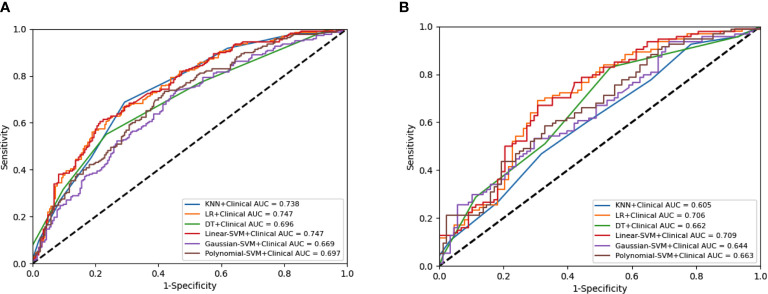
Receiver operating characteristic (ROC) curves of the combined model constructed by six algorithms in the training set **(A)** and internal test set **(B)**.

**Figure 6:**
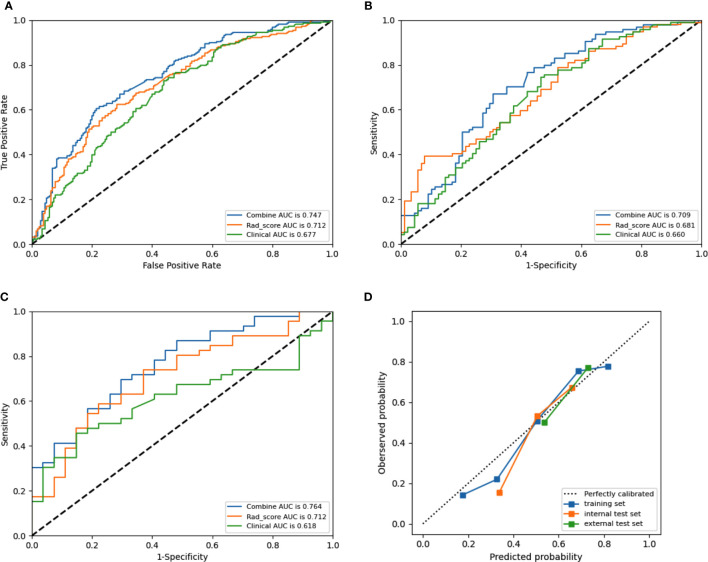
Receiver operating characteristic (ROC) curves of the combined model (blue lines), Rad-score model (orange lines) and clinical model (green lines) in the training set **(A)**, internal test set **(B)** set, and external test set **(C)**, respectively. Calibration curves **(D)** of the combined model in the training and test sets, respectively. The diagonal dotted line represents an ideal prediction, while the red line represents the performance of the training set, the blue line and black line represents the performance of the internal test set and external test set, respectively, Closer fit to the diagonal dotted line indicates a better prediction.

**Figure 7:**
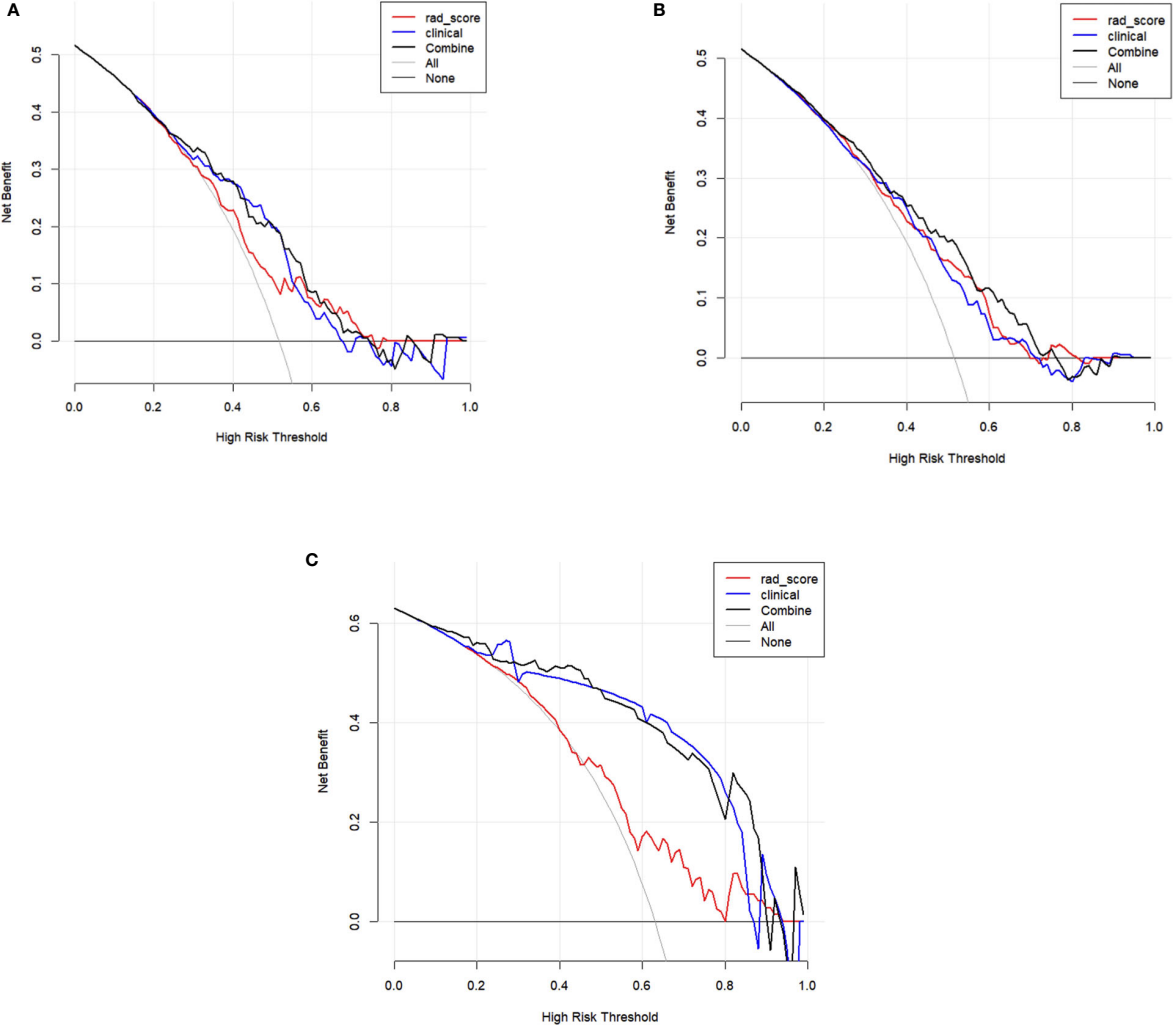
Decision curve analysis (DCA) of training set **(A)**, internal test set **(B)**, and external test set **(C)**. The y-axis measures the net benefit. The x-axis shows the corresponding risk threshold. The grey line represents the assumption that all lesions were central lymph node metastases. The black straight line represents the assumption that all nodules were non-CLNM. If the threshold probability was more than 30%, using the combined model (black line) to predict CLNM added more benefit than the Rad-score model (red line) and clinical model (blue line) in the internal test set; If the threshold probability was more than 10%, using the combined model (black line) to predict CLNM added more benefit than the Rad-score model (red line) and clinical model (blue line) in the external test set.

Tumor size, sex, age, tumor diameter, and CT-reported LN status were identified as independent clinical risk factors of CLNM in PTC. No collinearity was observed because the VIF of the predictor ranged from 1.10 to 1.25. The combined radiomics model included radiomic features and independent clinical risk factors. The performance of prediction among these six machine learning models in the training set and internal test set are detailed in **Figures 5** **A, B**. As shown in **Table 3**, in the internal test set, the highest AUC was 0.709 (95% CI 0.634–0.786) in linear-SVM, whereas the AUC values of the KNN, LR, DT, Gaussian-SVM, and polynomial-SVM were only 0.605 (95% CI 0.507–0.611), 0.706 (95% CI 0.610–0.725), 0.662 (95% CI 0.538–0.655), 0.644 (95% CI 0.520–0.630), and 0.663 (95% CI 0.533–0.647), respectively. In addition, the linear-SVM algorithm was also better than the CT reported LN status (experienced radiologist’s performance) in sensitivity (0.702 [95% CI 0.600–0.790] vs. 0.477 [95% CI 0.409–0.545]) and accuracy (0.670 [95% CI 0.600–0.738] vs. 0.642 [95% CI 0.569–0.712]) in the internal test set in the combined radiomics model. The combined radiomics model constructed using the linear-SVM algorithm performed the best for AUC 0.747 (95% CI 0.706–0.782) in the training set (**Figure 6** **A**), 0.709 (95% CI 0.634–0.786) in the internal test set (**Figure 6** **B**), and 0.764 (95%CI 0.654–0.875) in the external test set (**Figure 6** **C**). In the calibration curve (**Figure 6** **D**), the gray line represents perfect prediction, and the dotted line represents the calibration curve of the combined radiomics model. The calibration curve showed good agreement between the true state of central LN metastases and the predicted probability based on radiomics. The results of DCA are shown in **Figure 7**. When the threshold probability ranged from 0.2 to 0.7 in the internal test set and 0.1 to 0.9 in the external test set, the combined radiomics model to predict CLNM provides more net benefit than the “treat all” or “treat none” scheme. Therefore, this combined radiomics model constructed by linear-SVM showed excellent performance in discrimination, calibration, and clinical usefulness.

**Table 3:** The performance of combined models constructed by six algorithms in the internal test set.

	AUC	95%CI	SEN	95%CI	SPE	95%CI	ACC	95%CI
KNN+ Clinical	0.605	0.507-0.611	0.777	0.677-0.853	0.341	0.245-0.451	0.566	0.491-0.639
LR+ Clinical	0.706	0.610-0.725	0.723	0.620-0.808	0.614	0.503-0.714	0.670	0.570-0.738
DT+ Clinical	0.662	0.538-0.655	0.511	0.406-0.614	0.670	0.561-0.765	0.588	0.513-0.660
Lin-SVM+ Clinical	0.709	0.634-0.786	0.702	0.600-0.790	0.636	0.526-0.734	0.670	0.600-0.738
Gaus-SVM+ Clinical	0.644	0.520-0.630	0.766	0.665-0.845	0.386	0.286-0.497	0.582	0.507-0.645
Ploy-SVM+ Clinical	0.663	0.533-0.647	0.691	0.587-0.780	0.489	0.381-0.597	0.593	0.518-0.666
Clinical	0.660	0.570-0.786	0.574	0.468-0.674	0.636	0.526-0.734	0.604	0.530-0.676

KNN, K-nearest neighbor; LR, logistic regression; DT, decision tree; Lin-SVM, linear-support vector machine; Gaus-SVM, Gaussian- support vector machine; Ploy-SVM, polynomial-SVM.

## Discussion

Due to there’s still controversial in surgery strategies for thyroid carcinoma, preoperative prediction of thyroid cancer and LN status is of vital importance for the precise treatment of thyroid carcinoma (39). The judgement of LN status is the core factor for routine central LN dissection (40–43). Thus, noninvasive LN assessment tool is warranted. In this multicenter retrospective study, a CT-based comprehensive radiomics model including clinical risk factors and radiomic features was developed for the pretreatment prediction of CLNM in patients with PTC. The linear-SVM model demonstrated the highest performance of predicting CLNM, with an AUC of 0.764 in the external test set. Thus, the linear-SVM algorithm was chosen as the best and compared with experienced radiologist’s performance. The application of the combined radiomics model provides a new approach for establishing prediction models with multiple characteristics. This individualized model has satisfied the requirements of precision medicine development and can be used by doctors and patients easily.

Many studies have been conducted on this topic (44–48). Zhu et al. (46) applied six machine learning algorithms coupled with preoperative clinical characteristics and intraoperative information to develop prediction models for CLNM. Yang et al. (47) developed a multicenter nomogram with internal and external validation set C-index values of 0.854 and 0.825, respectively. However, their internal validation set was through a bootstrapping analysis, which might cause the overfitting of the model. In addition, these studies solely used clinical information to construct predictive models. By contrast, our multicenter research included an independent internal test set and an external test set and provided additional radiomics evidence in predicting the CLNM of PTC. Compared with US signature-based nomograms (45, 49, 50), our CT-based combined radiomics model, given the high clarity of CT images in identifying LN metastases, incorporated the CT-reported LN status, clinical risk factors, and radiomics features, which could be obtained through a non-invasive method before surgery. This combined radiomics model showed acceptable performance in predicting LN metastases.

This study offers other notable advantages. First, six combined radiomics models were constructed by machine learning algorithms (KNN, LR, DT, linear-SVM, Gaussian-SVM, and polynomial-SVM). The reliability and accuracy of the models can be better demonstrated by comparing the performance of these models. Linear-SVM showed the highest value among these six algorithms. The linear-SVM classifier is implemented specifically for massive levels of data and features (51). Relative to the KNN, LR, Gaussian-SVM, polynomial-SVM, and DT, linear-SVM has faster training and classification speed because it is well suited for high-dimensional features (52). In the present study, linear-SVM showed the highest classification accuracy (0.709), which is higher than the recognition accuracy of the KNN, LR, Gaussian-SVM, polynomial-SVM, and DT algorithms. Hence, the linear-SVM has prominent advantages over the other five algorithms in this study. Second, some preprocessing techniques were applied prior to feature extraction to improve feature discrimination. Third, this study is a multicenter study, and CT images were extracted from different data centers and different machines, which improved the generalization ability of the combined radiomics model.

This study has several limitations. First, our sample size was modest and only carried out in China, especially for the testing set, which may limit the performance of our predictive modelling. A large-scale, cross-race study is needed to improve the prediction efficiency of the model. Second, although the manual segmentation method has achieved satisfactory inter- and intra-observer reproducibility of radiomics feature extraction, over 90% of features have good reproducibility (53). However, the automated method of image segmentation may have high stability. Third, tumor diameters < 0.5 cm were not included because they could not be reliably identified and segmented on CT images. In the future, we will use smaller slice gaps and more advanced CT software to improve the detection of smaller tumors. Fourth, this study only analyzed single lesion tumor in patients with PTC instead of multiple tumors to avoid selection bias. Thus, the generalization ability of the model is limited. Fifth, the radiomic features were extracted from the primary tumor instead of LNs, which may affect the accuracy of the model. Moreover, only the conventional machine learning radiomics method was used in this study. Some studies have shown that deep learning methods have a certain value in predicting LN metastases. We will continue our research with deep learning methods in the future.

In summary, our combined radiomics model is a noninvasive predictive tool that combines the radiomic features of CT images with independent clinical risk factors and shows good application prospects. A large sample size, cross-race study should be carried out to optimize the model and improve the efficiency of the model in subsequent studies.

## Data Availability Statement

The original contributions presented in the study are included in the article/supplementary material. Further inquiries can be directed to the corresponding authors.

## Ethics Statement

Written informed consent was obtained from the individual(s) for the publication of any potentially identifiable images or data included in this article.

## Author Contributions

JL implemented the conceptualization, methodology, data curation, and writing original draft preparation. XW implemented literature searching, and manuscript writing. NM Identified the radiological characteristics of PTC and estimated and adjusted the accuracy of VOIs. GZ implemented VOI segmentation, and literature searching. HZ implemented data analysis and figures making. YM and CJ implemented visualization, and investigation. JM implemented writing- reviewing and editing. XS conducted the design, quality control, and data interpretation. All authors contributed to the article and approved the submitted version.

## Funding

This work was supported by the “Taishan Scholar” Project (No. ts20190991).

## Conflict of Interest

The authors declare that the research was conducted in the absence of any commercial or financial relationships that could be construed as a potential conflict of interest.

## Publisher’s Note

All claims expressed in this article are solely those of the authors and do not necessarily represent those of their affiliated organizations, or those of the publisher, the editors and the reviewers. Any product that may be evaluated in this article, or claim that may be made by its manufacturer, is not guaranteed or endorsed by the publisher.

## References

[1] Rosenbaum MA McHenry CR. Contemporary Management of Papillary Carcinoma of the Thyroid Gland. Expert Rev Anticancer Ther (2009) 9(3):317–29. doi: 10.1586/14737140.9.3.317 19275510

[2] Lim H Devesa SS Sosa JA Check D Kitahara CM. Trends in Thyroid Cancer Incidence and Mortality in the United States, 1974-2013. JAMA (2017) 317(13):1338. doi: 10.1001/jama.2017.2719 28362912 PMC8216772

[3] Abdullah MI Junit SM Ng KL Jayapalan JJ Karikalan B Hashim OH. Papillary Thyroid Cancer: Genetic Alterations and Molecular Biomarker Investigations. Int J Med Sci (2019) 16(3):450–60. doi: 10.7150/ijms.29935 PMC6428975 30911279

[4] Sawka AM Carty SE Haugen BR Hennessey JV Kopp PA Pearce EN. American Thyroid Association Guidelines and Statements: Past, Present, and Future: American Thyroid Association Guidelines Policy and Procedures Task Force. Thyroid (2018) 28(6):692–706. doi: 10.1089/thy.2018.0070 29698130

[5] Onuma AE Beal EW Nabhan F Hughes T Farrar WB Phay J. Long-Term Efficacy of Lymph Node Reoperation for Persistent Papillary Thyroid Cancer: 13-Year Follow-Up. Ann Surg Oncol (2019) 26(6):1737–43. doi: 10.1245/s10434-019-07263-5 PMC6511284 30820785

[6] Stack BC Ferris RL Goldenberg D Haymart M Shaha A Sheth S. American Thyroid Association Consensus Review and Statement Regarding the Anatomy, Terminology, and Rationale for Lateral Neck Dissection in Differentiated Thyroid Cancer. Thyroid (2012) 22(5):501–8. doi: 10.1089/thy.2011.0312 22435914

[7] Yeh MW Bauer AJ Bernet VA Ferris RL Loevner LA Mandel SJ. American Thyroid Association Statement on Preoperative Imaging for Thyroid Cancer Surgery. Thyroid (2015) 25(1):3–14. doi: 10.1089/thy.2014.0096 25188202 PMC5248547

[8] Durante C Montesano T Torlontano M Attard M Monzani F Tumino S. Papillary Thyroid Cancer: Time Course of Recurrences During Postsurgery Surveillance. J Clin Endocrinol Metab (2013) 98(2):636–42. doi: 10.1210/jc.2012-3401 23293334

[9] Tufano RP Clayman G Heller KS Inabnet WB Kebebew E Shaha A. Management of Recurrent/Persistent Nodal Disease in Patients With Differentiated Thyroid Cancer: A Critical Review of the Risks and Benefits of Surgical Intervention Versus Active Surveillance. Thyroid (2015) 25(1):15–27. doi: 10.1089/thy.2014.0098 25246079

[10] Chen L Zhu Y Zheng K Zhang H Guo H Zhang L. The Presence of Cancerous Nodules in Lymph Nodes is a Novel Indicator of Distant Metastasis and Poor Survival in Patients With Papillary Thyroid Carcinoma. J Cancer Res Clin Oncol (2017) 143(6):1035–42. doi: 10.1007/s00432-017-2345-2 PMC11819005 28204971

[11] Hughes DT Doherty GM. Central Neck Dissection for Papillary Thyroid Cancer. Cancer Control (2011) 18(2):83–8. doi: 10.1177/107327481101800202 21451450

[12] Sivanandan R Soo KC. Pattern of Cervical Lymph Node Metastases From Papillary Carcinoma of the Thyroid: Cervical Metastases From Papillary Thyroid Carcinoma. Br J Surg (2001) 88(9):1241–4. doi: 10.1046/j.0007-1323.2001.01843.x 11531874

[13] Mirallié E Visset J Sagan C Hamy A Bodic M-FL Paineau J. Localization of Cervical Node Metastasis of Papillary Thyroid Carcinoma. World J Surg (1999) 23(9):970–4. doi: 10.1007/s002689900609 10449830

[14] Al Afif A Williams BA Rigby MH Bullock MJ Taylor SM Trites J. Multifocal Papillary Thyroid Cancer Increases the Risk of Central Lymph Node Metastasis. Thyroid (2015) 25(9):1008–12. doi: 10.1089/thy.2015.0130 26161997

[15] Cabanillas ME McFadden DG Durante C. Thyroid Cancer. Lancet (2016) 388(10061):2783–95. doi: 10.1016/S0140-6736(16)30172-6 27240885

[16] Cady B. Our Ames is True: How an Old Concept Still Hits the Mark: or, Risk Group Assignment Points the Arrow to Rational Therapy Selection in Differentiated Thyroid Cancer. Am J Surg (1997) 174(5):462–8. doi: 10.1016/S0002-9610(97)00162-1 9374215

[17] Shaha AR. Implications of Prognostic Factors and Risk Groups in the Management of Differentiated Thyroid Cancer. Laryngoscope (2004) 114: (3):393–402. doi: 10.1097/00005537-200403000-00001 15091208

[18] Jeong H-S Baek C-H Son Y-I Choi J-Y Kim H-J Ko Y-H. Integrated ^18^ F-FDG PET/CT for the Initial Evaluation of Cervical Node Level of Patients With Papillary Thyroid Carcinoma: Comparison With Ultrasound and Contrast-Enhanced CT. Clin Endocrinol (2006) 65(3):402–7. doi: 10.1111/j.1365-2265.2006.02612.x 16918964

[19] Choi JS Kim J Kwak JY Kim MJ Chang HS Kim E-K. Preoperative Staging of Papillary Thyroid Carcinoma: Comparison of Ultrasound Imaging and CT. Am J Roentgenol (2012) 193:871–8. doi: 10.2214/AJR.09.2386 19696304

[20] Stulak JM. Value of Preoperative Ultrasonography in the Surgical Management of Initial and Reoperative Papillary Thyroid Cancer. Arch Surg (2006) 141(5):489. doi: 10.1001/archsurg.141.5.489 16702521

[21] Ito Y Tomoda C Uruno T Takamura Y Miya A Kobayashi K. Clinical Significance of Metastasis to the Central Compartment From Papillary Microcarcinoma of the Thyroid. World J Surg (2006) 30(1):91–9. doi: 10.1007/s00268-005-0113-y 16369721

[22] O’Connell K Yen TW Quiroz F Evans DB Wang TS. The Utility of Routine Preoperative Cervical Ultrasonography in Patients Undergoing Thyroidectomy for Differentiated Thyroid Cancer. Surgery (2013) 154(4):697–703. doi: 10.1016/j.surg.2013.06.040 24011674

[23] Kim E Park JS Son K-R Kim J-H Jeon SJ Na DG. Preoperative Diagnosis of Cervical Metastatic Lymph Nodes in Papillary Thyroid Carcinoma: Comparison of Ultrasound, Computed Tomography, and Combined Ultrasound With Computed Tomography. Thyroid (2008) 18(4):411–8. doi: 10.1089/thy.2007.0269 18358074

[24] Suh CH Baek JH Choi YJ Lee JH. Performance of CT in the Preoperative Diagnosis of Cervical Lymph Node Metastasis in Patients With Papillary Thyroid Cancer: A Systematic Review and Meta-Analysis. AJNR Am J Neuroradiol (2017) 38(1):154–61. doi: 10.3174/ajnr.A4967 PMC7963646 27789450

[25] Lee Y Kim J Baek JH Jung SL Park S-W Kim J. Value of CT Added to Ultrasonography for the Diagnosis of Lymph Node Metastasis in Patients With Thyroid Cancer. Head Neck (2018) 40(10):2137–48. doi: 10.1002/hed.25202 29756249

[26] Lambin P Rios-Velazquez E Leijenaar R Carvalho S van Stiphout RGPM Granton P. Radiomics: Extracting More Information From Medical Images Using Advanced Feature Analysis. Eur J Cancer (2012) 48(4):441–6. doi: 10.1016/j.ejca.2011.11.036 PMC4533986 22257792

[27] Gillies RJ Kinahan PE Hricak H. Radiomics: Images Are More Than Pictures, They Are Data. Radiology (2016) 278(2):563–77. doi: 10.1148/radiol.2015151169 PMC4734157 26579733

[28] Wu L Yang X Cao W Zhao K Li W Ye W. Multiple Level CT Radiomics Features Preoperatively Predict Lymph Node Metastasis in Esophageal Cancer: A Multicentre Retrospective Study. Front Oncol (2020) 9:1548. doi: 10.3389/fonc.2019.01548 32039021 PMC6985546

[29] Ji G-W Zhang Y-D Zhang H Zhu F-P Wang K Xia Y-X. Biliary Tract Cancer at CT: A Radiomics-Based Model to Predict Lymph Node Metastasis and Survival Outcomes. Radiology (2019) 290(1):90–8. doi: 10.1148/radiol.2018181408 30325283

[30] Zhang W Fang M Dong D Wang X Ke X Zhang L. Development and Validation of a CT-Based Radiomic Nomogram for Preoperative Prediction of Early Recurrence in Advanced Gastric Cancer. Radiother Oncol (2020) 145:13–20. doi: 10.1016/j.radonc.2019.11.023 31869677

[31] Liu T Zhou S Yu J Guo Y Wang Y Zhou J. Prediction of Lymph Node Metastasis in Patients With Papillary Thyroid Carcinoma: A Radiomics Method Based on Preoperative Ultrasound Images: Technology in Cancer Research & Treatment. Technol Cancer Res Treat (2019) 18:1533033819831713. doi: 10.1177/1533033819831713 30890092 PMC6429647

[32] Yu J Deng Y Liu T Zhou J Jia X Xiao T. Lymph Node Metastasis Prediction of Papillary Thyroid Carcinoma Based on Transfer Learning Radiomics. Nat Commun (2020) 11(1):4807. doi: 10.1038/s41467-020-18497-3 32968067 PMC7511309

[33] Haddad RI Kandeel F Scheri RP. NCCN Guidelines Index Table of Contents Discussion. (2019) 132:. doi: 10.1016/S0090-4295(19)30765-4

[34] Liu X Ouyang D Li H Zhang R Lv Y Yang A. Papillary Thyroid Cancer: Dual-Energy Spectral CT Quantitative Parameters for Preoperative Diagnosis of Metastasis to the Cervical Lymph Nodes. Radiology (2015) 275(1):167–76. doi: 10.1148/radiol.14140481 25521777

[35] Randolph GW Duh Q-Y Heller KS LiVolsi VA Mandel SJ Steward DL. The Prognostic Significance of Nodal Metastases From Papillary Thyroid Carcinoma Can Be Stratified Based on the Size and Number of Metastatic Lymph Nodes, as Well as the Presence of Extranodal Extension. Thyroid (2012) 22(11):1144–52. doi: 10.1089/thy.2012.0043 23083442

[36] Zhao Y Li X Li L Wang X Lin M Zhao X. Preliminary Study on the Diagnostic Value of Single-Source Dual-Energy CT in Diagnosing Cervical Lymph Node Metastasis of Thyroid Carcinoma. J Thorac Dis (2017) 9(11):4758–4766–4766. doi: 10.21037/jtd.2017.09.151 PMC5721007 29268547

[37] Landis JR Koch GG. The Measurement of Observer Agreement for Categorical Data. Biometrics (1977) 33(1):159–74. doi: 10.2307/2529310 843571

[38] Platt J. Probabilistic Outputs for Support Vector Machines and Comparisons to Regularized Likelihood Methods. Adv Large Margin Classifiers (1999) 10(3):61–74.

[39] Conzo G Calò PG Gambardella C Tartaglia E Mauriello C Della Pietra C. Controversies in the Surgical Management of Thyroid Follicular Neoplasms. Retrospective Anal 721 Patients Int J Surg (2014) 12 Suppl 1:S29–34. doi: 10.1016/j.ijsu.2014.05.013 24859409

[40] Haugen BR Alexander EK Bible KC Doherty GM Mandel SJ Nikiforov YE. 2015 American Thyroid Association Management Guidelines for Adult Patients With Thyroid Nodules and Differentiated Thyroid Cancer: The American Thyroid Association Guidelines Task Force on Thyroid Nodules and Differentiated Thyroid Cancer. Thyroid (2016) 26(1):1–133. doi: 10.1089/thy.2015.0020 26462967 PMC4739132

[41] Viola D Materazzi G Valerio L Molinaro E Agate L Faviana P. Prophylactic Central Compartment Lymph Node Dissection in Papillary Thyroid Carcinoma: Clinical Implications Derived From the First Prospective Randomized Controlled Single Institution Study. J Clin Endocrinol Metab (2015) 100(4):1316–24. doi: 10.1210/jc.2014-3825 25590215

[42] Hartl DM Leboulleux S Al Ghuzlan A Baudin E Chami L Schlumberger M. Optimization of Staging of the Neck With Prophylactic Central and Lateral Neck Dissection for Papillary Thyroid Carcinoma. Ann Surg (2012) 255(4):777–83. doi: 10.1097/SLA.0b013e31824b7b68 22418010

[43] Conzo G Docimo G Ruggiero R Napolitano S Palazzo A Gambardella C. Surgical Treatment of Papillary Thyroid Carcinoma Without Lymph Nodal Involvement. G Chir (2012) 33(10):339–42. 23095564

[44] Zhao W He L Zhu J Su A. A Nomogram Model Based on the Preoperative Clinical Characteristics of Papillary Thyroid Carcinoma With Hashimoto’s Thyroiditis to Predict Central Lymph Node Metastasis. Clin Endocrinol (2021) 94(2):310–21. doi: 10.1111/cen.14302 32984984

[45] Zhou SC Liu TT Zhou J Huang YX Guo Y Yu JH. An Ultrasound Radiomics Nomogram for Preoperative Prediction of Central Neck Lymph Node Metastasis in Papillary Thyroid Carcinoma. Front Oncol (2020) 10:1591. doi: 10.3389/fonc.2020.01591 33014810 PMC7498535

[46] Zhu J Zheng J Li L Huang R Ren H Wang D. Application of Machine Learning Algorithms to Predict Central Lymph Node Metastasis in T1-T2, Non-Invasive, and Clinically Node Negative Papillary Thyroid Carcinoma. Front Med (2021) 8:635771. doi: 10.3389/fmed.2021.635771 PMC7986413 33768105

[47] Yang Z Heng Y Lin J Lu C Yu D Tao L. Nomogram for Predicting Central Lymph Node Metastasis in Papillary Thyroid Cancer: A Retrospective Cohort Study of Two Clinical Centers.. Cancer Res Treat (2020) 52:1010–8. doi: 10.4143/crt.2020.254 PMC7577812 32599980

[48] Kim SK Chai YJ Park I Woo JW Lee JH Lee KE. Nomogram for Predicting Central Node Metastasis in Papillary Thyroid Carcinoma: Nomogram for Predicting CLNM in PTC. J Surg Oncol (2017) 115(3):266–72. doi: 10.1002/jso.24512 27859312

[49] Huang C Cong S Liang T Feng Z Gan K Zhou R. Development and Validation of an Ultrasound-Based Nomogram for Preoperative Prediction of Cervical Central Lymph Node Metastasis in Papillary Thyroid Carcinoma. Gland Surg (2020) 9(4):956–67. doi: 10.21037/gs-20-75 PMC7475364 32953605

[50] Tian X Song Q Xie F Ren L Zhang Y Tang J. Papillary Thyroid Carcinoma: An Ultrasound-Based Nomogram Improves the Prediction of Lymph Node Metastases in the Central Compartment. Eur Radiol (2020) 30(11):5881–93. doi: 10.1007/s00330-020-06906-6 32588211

[51] Fan R-E Chang K-W Hsieh C-J Wang X-R Lin C-J. LIBLINEAR: A Library for Large Linear Classification. J Mach Learn Res (2008) 9:1871–4.

[52] Yang W Si Y Wang D Guo B. Automatic Recognition of Arrhythmia Based on Principal Component Analysis Network and Linear Support Vector Machine. Comput Biol Med (2018) 101:22–32. doi: 10.1016/j.compbiomed.2018.08.003 30098452

[53] Lee M Woo B Kuo MD Jamshidi N Kim JH. Quality of Radiomic Features in Glioblastoma Multiforme: Impact of Semi-Automated Tumor Segmentation Software. Korean J Radiol (2017) 18(3):498–509. doi: 10.3348/kjr.2017.18.3.498 28458602 PMC5390619

